# Photonic Band Gap and Bactericide Performance of Amorphous Sol-Gel Titania: An Alternative to Crystalline TiO_2_

**DOI:** 10.3390/molecules23071677

**Published:** 2018-07-10

**Authors:** M. Clara Gonçalves, José Carlos Pereira, Joana C. Matos, Helena Cristina Vasconcelos

**Affiliations:** 1 Departamento de Engenharia Química, Instituto Superior Técnico, Universidade de Lisboa, Av. Rovisco Pais, 1049-001 Lisboa, Portugal; jose.carlos.pereira@tecnico.ulisboa.pt; 2 CQE, Centro de Química Estrutural, Instituto Superior Técnico, Universidade de Lisboa, 1049-001 Lisboa, Portugal; joana.matos@tecnico.ulisboa.pt; 3 Faculty of Sciences and Technology, Azores University, Ladeira da Mãe de Deus, 9501-855 Ponta Delgada, Portugal; hcsv@uac.pt; 4 Centre of Physics and Technological Research (CEFITEC), FCT/UNL Faculdade de Ciências e Tecnologia, 2829-516 Caparica, Portugal

**Keywords:** amorphous-TiO_2_, photonic band-gap, bactericide, NPs, films, sol-gel, *E. coli*

## Abstract

In addition to its traditional application in white pigments, nanocrystalline titania (TiO_2_) has optoelectronic and photocatalytic properties (strongly dependent on crystallinity, particle size, and surface structure) that grant this naturally occurring oxide new technological applications. Sol-gel is one of the most widely used methods to synthesize TiO_2_ films and NPs, but the products obtained (mostly oxy-hydrated amorphous phases) require severe heat-treatments to promote crystallization, in which control over size and shape is difficult to achieve. In this work, we obtained new photocatalytic materials based on amorphous titania and measured their electronic band gap. Two case studies are reported that show the enormous potential of amorphous titania as bactericide or photocatalyst. In the first, amorphous sol-gel TiO_2_ thin films doped with N (TiO_2−*x*_N*_x_*, *x =* 0.75) were designed to exhibit a photonic band gap in the visible region. The identification of Ti-O-N and N-Ti-O bindings was achieved by XPS. The photonic band gaps were found to be 3.18 eV for a-TiO_2_ and 2.99 eV for N-doped a-TiO_2_. In the second study, amorphous titania and amine-functionalized amorphous titania nanoparticles were synthetized using a novel base-catalysed sol-gel methodology. All the synthesized amorphous TiO_2_ nanoparticles exhibit bactericide performance (*E. coli*, ASTME 2149-13).

## 1. Introduction

Since its commercial production in the early 20th century, titanium dioxide (TiO_2_) has been used in metallurgy and in white pigments in formulations such as paints, toothpastes, ointments, cosmetics, and sunscreens, due to its intense whiteness and purity ([1] and references within). However, it was the discovery of photocatalytic splitting of water on a TiO_2_ electrode under ultraviolet light (UV) in 1972 [2] that brought TiO_2_ to the spotlight. Since then, much research has been focused on TiO_2_ photocatalysis, leading to promising applications in areas ranging from photovoltaics, photocatalysis, and electrochemistry. In addition, the surface of TiO_2_ exhibits high hydrophilicity under UV light irradiation with a water contact angle of 0° [3]. Water spitting, self-cleaning, anti-fogging, sterilization and disinfection, prevention of stains, lithography, photodegradation of organic pollutants or catalytic oxidation of carbon monoxide, metal corrosion prevention, sensoring are some of the actions that TiO_2_ can efficiently perform based on the strong oxidizing power of TiO_2_ under UV radiation, enabling a wide range of industrial applications [4,5,6,7,8,9,10,11].

Amorphous-TiO_2_ has raised great interest in the academic community, in which a set of a-TiO_2_ applications has been proved: as a high performance photocatalyst [12,13], as dye sensitizer [14] or electrode [15] in solar batteries, as thin film in capacitors [16], in resistive random access memory applications [17], as a self-cleaning agent due to its super-hydrophylicity [18,19,20], or to purify dye-polluted water [21]. Recent applications include anodes for sodium ion rechargeable batteries [22], low temperature oxygen sensors [23], visible light photocatalysts [13,24], or biomedical applications [25,26].

### 1.1. Titania Structure

**Figure 1:**
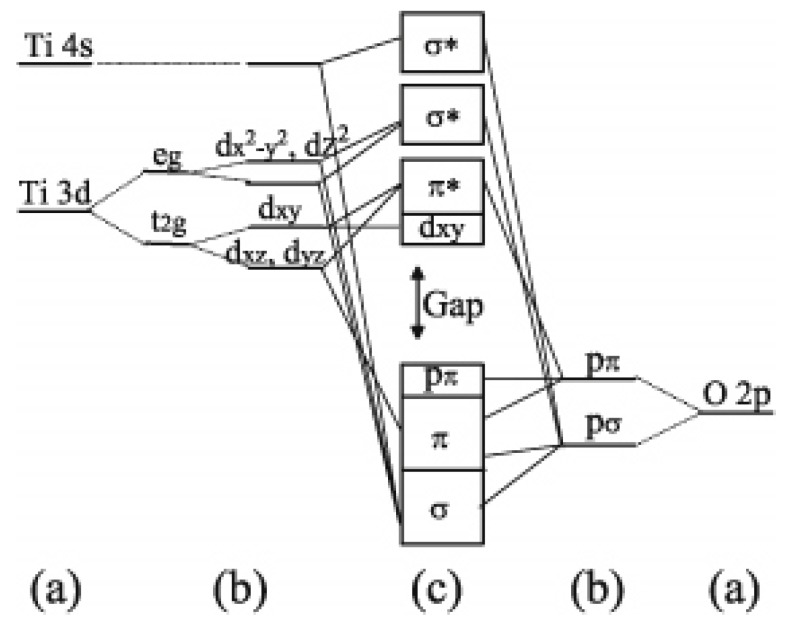
Electronic band structures of rutile and anatase. (a) Atomic levels; (b) crystal-filed split levels; and (c) final interactions states. The thin solid and dashed lines represent large and small contributions, respectively. (Reprint with permission from American Physical Society: Asahi R.; Taga Y.; Mannstadt W.; Freeman A. Electronic and Optical Properties of Anatase TiO_2_. J Phys Review B 2000, 61, 7459, [28]).

Titania occurs in nature in various crystalline phases, being the most common anatase (tetragonal), rutile (tetragonal), brookite (rhombohedral), and TiO_2_(B) (monoclinic) [1,11,27]. Rutile and anatase, the more studied and used crystalline phases industrially, share the same building block, a TiO_6_ octahedron. Rutile forms linear chains in which each octahedron is linked to 10 other octahedra (two sharing edges and eight sharing corners), while anatase forms zigzag chains with a screw axis in which each octahedron is linked to 8 other octahedra (four sharing edges and four sharing corners). In anatase, the Ti-Ti distances are larger and the Ti-O distances are smaller than in rutile. A slight orthorhombic distortion is observed in rutile and even more in anatase. These differences in structure originate different densities and electronic band structures for the two TiO_2_ polymorphs (Figure 1).

**Figure 2:**
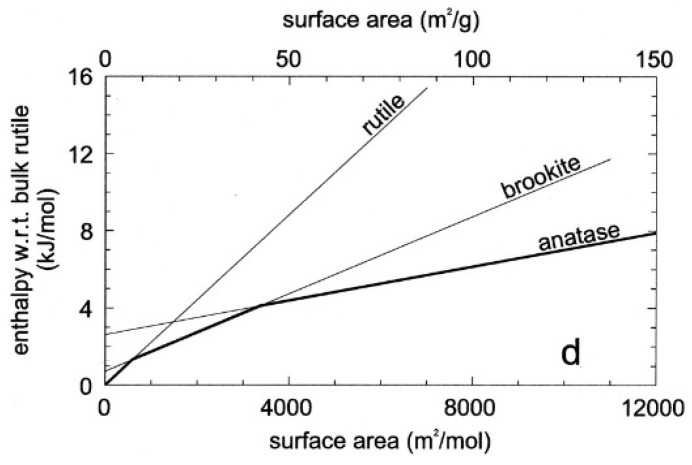
Anatase and rutile thermodynamic stability. (Reprint with permission from Ranade M.R.; Navrotsky A.; Zhang H.Z.; Banfield J.F.; Elder S.H.; Zaban A.; Borse P.H.; Kulkarmi S.K.; Doran G.S.; Whitfield H.J. Proc Natl Acad Sci USA 2002, 99, 6476, [35]. Copyright (2002) National Academy of Sciences, Washington, WA, USA.

Anatase, brookite, and TiO_2_(B) (all metastable phases) will go through phase transformation at elevated temperatures—anatase and brookite to rutile, anatase to brookite to rutile, and brookite and TiO_2_(B) to anatase to rutile [1,11,27]. Anatase may change to amorphous phases rather than to rutile under some experimental conditions, for example, in high energy milling operations [29] or during crystallite refinement [30]. At macro and micro scales, rutile is the thermodynamically stable phase under ambient conditions, although it is anatase that first crystallizes in many synthetic routes, due to a more flexible assembly of 4-edge-sharing TiO_6_ octahedron and crystal growth conditions [31,32,33]. At nanoscale, the phase thermodynamically more stable is anatase for nanoparticles (NP) smaller than 11 nm, brookite for NP sizes between 11 and 35 nm, and rutile for NPs larger than 35 nm [30,34] (Figure 2).

Amorphous TiO_2_ (a-TiO_2_) can be produced in bulk, films, or NPs using CVD or wet chemistry methods, namely, using sol-gel processes, which are the most widely used. For a-TiO_2_ NPs, a consensual view has not yet been reached for the atomic structure. The model that best fits the experimental results seems to be a slightly distorted, anatase-like crystalline core (with at least 2 unit cells) surrounded by an outer distorted shell with approximately 2–4 atomic layers (of octahedral-like structure), with coordination numbers Z_Ti-O_ = 5.3 and Z_O-Ti_ = 1.94 [36,37,38]. While the NPs crystalline core is essentially constant, the structure of the shell depends strongly on its size. A great number of structural defects, including under-coordinated sites (like TiO_3_, TiO_4_, or TiO_5_) and dangling bonds, are present in the shell and play a determinant role in the properties of a-TiO_2_ NPs [25]. Atomistic modelling simulations also predict a distorted octahedral structure for the core (with Z_Ti-O_~6.0 and Z_O-Ti_~3.0), with a more porous structure for the shell, with a large number of structural defects (with Z_Ti-O_ ≠ 6.0 and Z_O-Ti_ ≠ 3.0) [39,40] and a density value (3.74 g/cm^3^) lower than for bulk anatase (3.90 g/cm^3^).

### 1.2. Titania Band Gap

The general principles determining the electronic structure in crystalline solids are well established, both theoretically and experimentally. For solids (usually metals) with outer valence electrons, which are essentially unbounded to specific atomic nuclei, the experimental evidence (electrical and thermal properties) is well supported by the so-called *Free Electron* model, in which only kinetic energy is considered for those electrons. The quantified levels of energy occupied by these electrons are very close, forming an essentially continuous region of allowed energies. Due to the *Pauli Exclusion Principle*, the electrons occupy levels successively higher, until all electrons are attributed, defining the *Fermi* energy, E_F_, that changes very little with temperature. This model explains well the negligible contribution of the free electrons for the specific heat and provides results for electrical and thermal conductivities that are in agreement with the experimental results for most metals.

For crystalline systems, in which interactions between the periodic potential of the nuclei and the outer electrons must be considered, band theory models (*Kronning-Penney Nearly-Free Electron*, *Tight-Binding Approximation* [41,42]) show that large regions of energy are no longer available for the outer electrons, effectively creating gaps of forbidden energy, E_g_. This is confirmed experimentally by light absorption measurements, which show a sharp increase in absorption when the incident photons reach the energy required to transfer electrons across the gap to the higher empty levels [41]. The electronic gap observed in crystalline solids is influenced by the atomic structure. For instance, anatase has a band gap of 3.23 eV, while rutile, a more ordered crystalline structure, has a narrower band gap of 3.0 eV [1].

The electronic structure of crystalline systems is usually described as dividing the wave space in *Brillouin* zones. The borders of these *Brillouin* zones tend to have discontinuities in energy that cause the band gaps [41,43]. Therefore, these gaps separate regions of energy, one for each *Brillouin* zone, which can be associated with bands of allowed energy.

The physical constructions and mathematical tools developed for crystalline solids, such as reciprocal space, *Brillouin* zones, and *Bloch* functions, are no longer available for amorphous solids. Therefore, the models predicting and explaining the occurrence of energy gaps in crystals cannot be applied in amorphous solids.

However, a large amount of experimental evidence and theoretical work [44,45,46,47,48] seem to show that an electronic gap still occurs in amorphous solids. The sharp features in the density of states of crystalline solids [42,48] are no longer observed, but a region of forbidden energy still exists [44,48]. The emerging wisdom is that when short-range interactions between electrons are dominant (usually in covalent and ionic bonding), the electronic density of states is mainly determined by the atomic short range order [48]. Many amorphous solids have the same short-range order as the corresponding crystals (lacking it progressively, for example, due to dihedral angle disorder [49]), so they should have a similar gap of energy. Amorphous titania in its various reduced states can largely be viewed as having the same building blocks as in crystalline titania (Ti-O_n_ polyhedra, with *n* = 5, 6, 7) [50], but it lacks a long-range order, as these polyhedra are randomly oriented.

A range of Molecular Dynamics and Monte-Carlo simulations [36,40,51,52] seem to indicate that a-TiO_2_ should be formed by chains of Ti-O octahedra with coordination numbers Z_Ti-O_~5.5–6.0 and Z_O-Ti_~2.5–3.0 and a Ti-O bond length around 1.940 Å [36], close to what occurs in crystalline phases, thus supporting the thesis that a gap still exists in a-TiO_2_ in spite of the lack of long range order.

**Figure 3:**
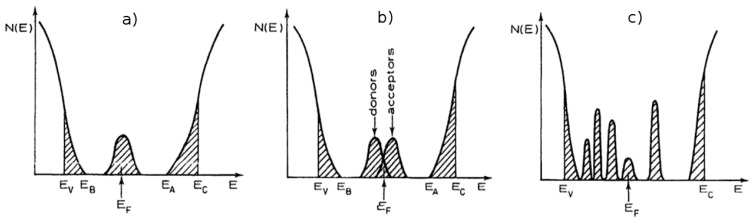
Density of states for amorphous solids: (**a**) the Davis-Mott model, showing a band of compensated levels near the middle of the gap; (**b**) the Marshall-Owen model with donor/acceptor bands in the middle of the gap; (**c**) a “real” glass with defect states. (Reprint with permission from Brodsky M.H. *Amorphous Semiconductors*, 2nd ed.; Springer-Verlag, Berlin, Germany, 1979, 357–393; ISBN. 978-0387160085, [53]).

The various models proposed [45,48] agree that in a crystalline to amorphous transition (called an *Anderson* transition [54]), some of the extended levels of energy (which are valid for the whole crystal, with a finite contribution for conductivity, described by *Bloch* wave functions) must be replaced by localized electron states, which can be described by wave functions that are confined to a few atoms. Away from the central atom, the wave function amplitude decreases exponentially as *exp(−a r)*, in which *a* is the inverse localization length. Therefore, these electrons cannot contribute significantly to conductivity [48]. These localized electron levels seem to be concentrated near the top E_V_ of the valence band and in the bottom E_C_ of the conduction band (see Figure 3), creating tails that extend the valence and conduction bands, reducing effectively the electronic band gap [55,56]. According to some experimental evidence, smaller gaps have been measured for successively more disordered systems [46].

*Ab-initio* calculations for a-TiO_2_, showing the similarity of shape of the electronic density of states in the conduction band of amorphous and crystalline TiO_2_ phases, have also been reported [50,57,58,59,60]. Ab initio DFT calculations of the electronic gap for a-TiO_2_ give results in the range 2.2–2.85 eV [50,60], which compare with the experimental values of 3.03 eV for rutile and 3.20 eV for anatase TiO_2_ [50], which are slightly larger than expected.

Most models [48,53] predict also the existence of additional bands of localized levels in the middle of the electronic gap, in which the *Fermi* energy is located. Unlike extrinsic semiconductors, in which the presence of electron donor or acceptor impurities changes the *Fermi* level at low temperatures, in amorphous solids the Fermi level remains fixed, approximately in the middle of the band gap. Real densities of states in amorphous materials are expected to include several of these bands of localized levels, spread over the entire electronic band gap [53].

According to the model of *David and Mott* [48], the tailing of the localized states into the band gap is just a fraction of an electron volt, so the amorphous optical gap E_A_–E_B_ is close to the crystalline gap E_C_–E_V_ (see Figure 3). A band of localized states in the middle of the band gap, where the *Fermi* level lies, is also predicted and does not change with impurities. At high temperatures, electron-hole pairs are created by excitation to extended levels, from E_V_ to E_C,_ and normal conductivity in the conduction/valence bands occurs (see Figure 3). Electrons occasionally get trapped by local levels E_D_ below E_C,_ causing the conductivity to decrease overall. At intermediate temperatures, electrons can only jump from the valence band edge to localized states in the tail above E_V_, so conduction occurs only via the hopping of electrons in the local states (and normal conduction by holes in the valence band). At low temperature, the most probable conduction mechanism is again electron hopping, in the localized band in which the *Fermi* level lies, through a mechanism called variable range hopping, in which the electrons’ tunnel variable ranges between successive localized states, and conductivity is quite low.

### 1.3. Titania Photocatalytic Activity

Despite numerous efforts, the mechanistic details of the TiO_2_ photocatalyzed reactions remain complex and not completely understood. Photocatalytic activity appears to be dependent on the nature of the reactants and the overall process, and on the catalyst surface, resulting from the competition of various elementary phenomena [61,62]. Anatase exhibits the highest photoactivity due to a slightly higher Fermi level and a combination of crystallinity, improved charge carrier mobility, and large specific surface area, providing a higher number of surface hydroxyl groups (acting in titania photocatalytic reactions) [6]. Rutile has a narrower band gap and lower photocatalytic activity.

TiO_2_ photocatalytic reactions are initiated when a photon (*hv*) of energy equal or higher than the semiconductor band gap is absorbed by the material. The photoexcited electron (*e*^−^) is promoted from the (filled) valence band (*VB*) to the (empty) conduction band (*CB*), creating a ‘*hole’* (*h*^+^) in the *VB*. Once an *electron-hole pair* (*e*^−^-*h*^+^) is created, three main mechanisms may occur:

(a) Recombination path: by far the most common outcome, in which ~90% of *e*^−^-*h*^+^ pairs quickly recombine without any chemical effect simply by dissipating the absorbed *hv* energy as heat [5,6].
(b) Trapping path: responsible for about ~8% of the events, in which one or both charges migrate to defects in the crystalline network [5,6]. Trapping makes the material a somewhat weaker reductant or oxidant, although it preserves the charge separation, thus allowing for future photocatalysis reactions.
(c) Reactional path: the fraction of photo-generated charges promoting chemical reactions with surface adsorbed molecules is considered to be responsible for only 1–2% of the events [5,6]. One or both charge carriers (*e*^−^, *h*^+^), after migrating to the surface, react with adsorbed molecules. As each redox reaction involves only one electron, the even number of valence electrons usually found in molecules will be upset, and radicals (with one unpaired electron) will form.

Oxidizing reactions are promoted by the strong oxidant power of *h*^+^ (which is able to strip one electron from an adsorbed molecule). Organic radicals form in direct reactions, while hydroxyl and superoxide anion radicals form in indirect ones:

(1) $$h^{+}+H_{2}O\to {}^{•}OH(ad)+H^{+}$$

(2) $$h^{+}+O_{2}^{-}(ad)\to 2{}^{•}O(ad)$$

^•^OH and ^•^O are very powerful oxidants, which oxidise (with rapid kinetics) almost any molecule they encounter. Reducing reactions are carried out by photoexcited *e*^−^. In this case, adsorbed organic molecules are reduced (by taking *e*^−^), and O_2_ (or H_2_O) form ^•^O_2_^−^:

(3) $$e^{-}+O_{2}\to O_{2}^{-}(ad)$$

(4) $$O_{2}^{-}(ad)+H^{+}\to HO_{2}^{•}(ad)$$

The superoxide radical anion engages in a series of reactions with water, oxygen, and additional *e*^−^, generating a cascade of reactive oxygen species (ROS) like ^•^HO_2_^−^, H_2_O_2,_ and ^•^OH^−^.

**Figure 4:**
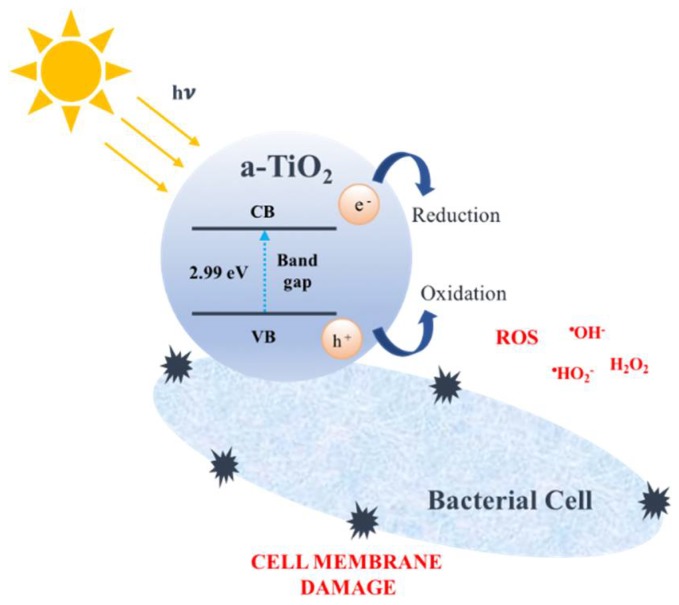
ROS damage bacterial cell membranes.

ROS play a pivotal role in sterilization and antimicrobial performance. When in close proximity to bacteria, ROS damage bacterial cell membranes through lipid peroxidation, enhance membrane fluidity, and cause disruption of the cell integrity, which is driven to cell death [63] (Figure 4). The required concentration of crystalline TiO_2_ NPs for killing bacteria varies in the range of 100–1000 ppm, depending on the NPs size, as well as the intensity and wavelength of the light source [5].

### 1.4. Titania Doping

**Figure 5:**
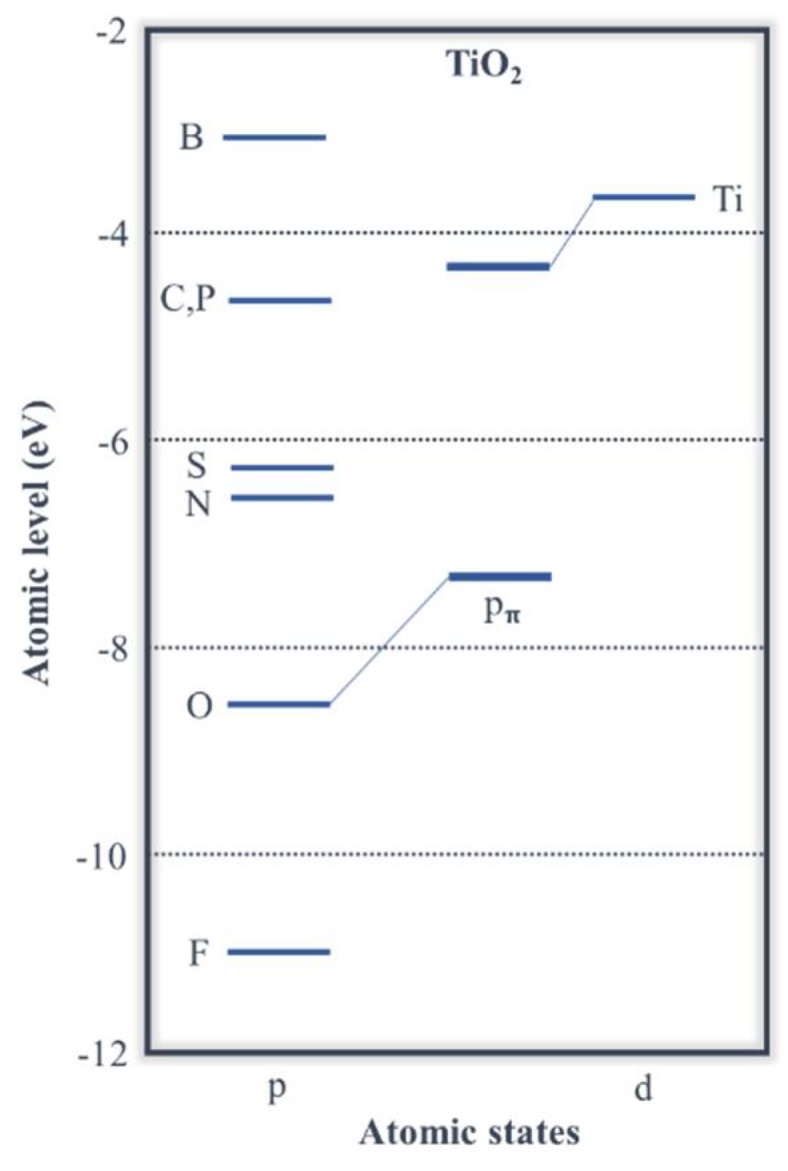
Comparison of atomic *p* levels of different anions (Adapted from [65]).

The increase in the sensitivity of TiO_2_ under visible light has been extensively studied, in order to expand the indoor/sunlight TiO_2_ applications. The addition of a visible-light sensitization dopant is a common strategy to decrease the rutile or anatase band gap, which is carried out by metal or non-metal elements. Adding atoms of other *3-d* transition metals introduces lower energy levels of metallic *d* states in the titania band gap, decreasing CB. When doping with non-metals, the non-bonding p_π_ state of O shallows or mixes with the states of the dopant anion, increasing VB. N is the element (in substitutional solid solution) that offers the most promising visible-light sensitizer opportunities for titania (Figure 5), replacing the dominant transitions (from O 2*_pπ_* to Ti d*_xy_*) at the absorption edge by those from N 2*_px_* to Ti d*_xy_* [64,65]. Small concentrations of N, doped under mild conditions, lead to interstitial doping, which also promotes the creation of oxygen vacancies. As the inclusion of N^3−^ is balanced by the elimination of O^2−^, for each 2 N incorporated 3 O^2−^ are removed, which increases the number of oxygen vacancies (*h*_O_^2−^) enhancing the photocatalysis activity.

To optimize photocatalysis performance, recombination and trapping processes, along with diffusional distances, should be minimized, an advantage for amorphous systems. The absence of phase boundaries in amorphous materials significantly decreases the trapping processes. a-TiO_2_ is often the first phase to form in many wet chemical synthetic routes (the first nuclei to form are anatase that rapidly evolve to oxy-hydroxy amorphous phases [36,37,38,66]). Amorphous TiO_2_ is also easier to process into different forms due to its lower order state, which permits a much wider range of dopants, and because it exhibits larger surface areas, with more -OH groups and more structural defects, enhancing the photocatalytic activity. Finally, it is much easier to remove oxygen from amorphous than from crystalline titania, resulting in more reaction sites on the a-TiO_2_ surface [50].

### 1.5. Titania Case Studies

In the present work, two case studies highlighting the photonic band gap and bactericide performance of a-TiO_2_, in thin films and NPs, are presented. In case study 1, amorphous sol-gel films of TiO_2−*x*_N*_x_* compositions, with N concentrations up to *x =* 0.75 (25 at*.*%), exhibit bandgaps almost identical to anatase, with photoabsorption spectra observed in the energy range 2.9–10.8 eV (320–115 nm). In case study 2, bactericide a-TiO_2_-based *NPs* are produced by sol-gel methodology under base-catalyzed conditions. Bactericide performance of a-TiO_2_-based NPs was proved according to ASTM E 2149-13 (*E. coli*). Sol-gel methodology was followed in both case studies. Sol-gel is one of the most widely used methods in the synthesis of a-TiO_2_ thin films and NPs, due to a huge number of advantages, like morphological and chemical homogeneity, high purity, low processing temperature, and wide process versatility [67,68].

## 2. Results and Discussion

### 2.1. Case Study 1. Band Gap of Amorphous TiO_2−x_N_x_ (x = 0; 0.75)

**Figure 6:**
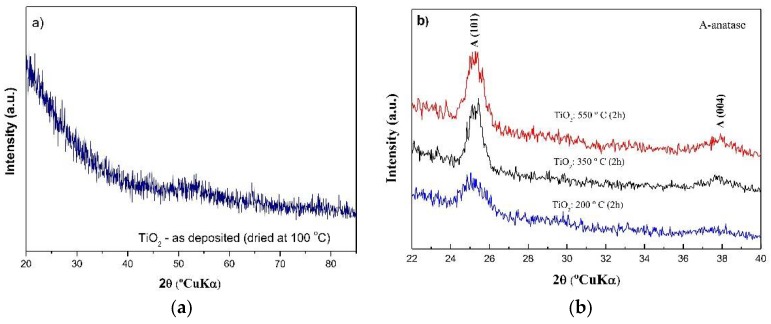
XRD patterns of pure TiO_2_ films: (**a**) as-deposited and (**b**) heat-treated (from 200 °C up to 550 °C).

X-ray diffraction measurements have been carried out for two kinds of samples: pure and N-doped sol-gel titania films (TiO_2−*x*_N*_x_*, respectively, with *x =* 0 and x = 0.75). The XRD analysis of the pure as-deposited TiO_2_ film showed an amorphous structure (Figure 6a).

**Figure 7:**
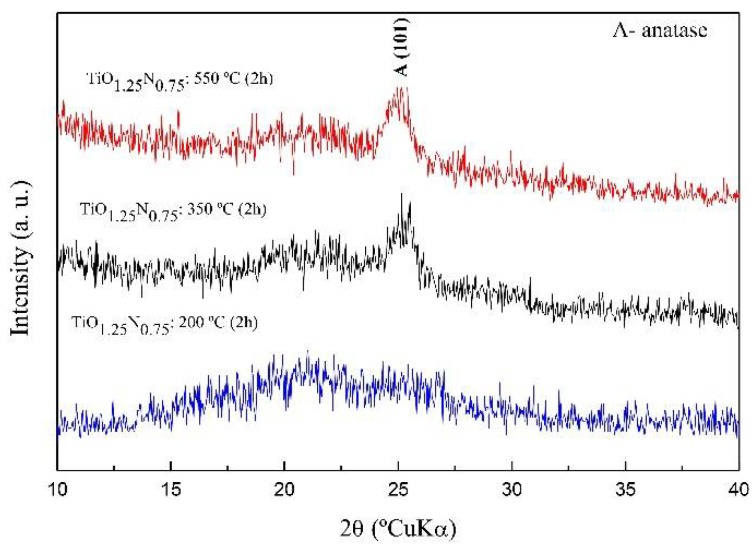
XRD patterns of TiO_0.25_N_0.75_ films after heat treatments (from 200 °C up to 550 °C).

The XRD patterns of pure TiO_2_ films after heat treatments (from 200 °C up to 550 °C) are shown in Figure 6b. For the lower temperature (200 °C), pure TiO_2_ films exhibited a dominant anatase reflection peak at 2θ~25.4° ({101} planes) with an additional minor reflection at 2θ~38° ({004} planes). The intensity of the anatase reflection peaks increases with temperature, up to 550 °C. No other crystalline phases were identified. In the case of doped TiO_2−*x*_N*_x_* films (Figure 7), no diffraction peaks are observed until 200 °C, indicating the inhibition of the amorphous → anatase phase transition due to N doping, in accordance with the work of Li et al. [69], who found that the incorporation of N in TiO_2_ films inhibits the crystallinity of TiO_2_.

At 350 °C, a weak and broad peak started to form, attributed to a dominant anatase phase (nanocrystals precipitation) in TiO_0.25_N_0.75_ amorphous matrix (the amorphous → anatase phase transition was delayed but not inhibited) in accordance with Li et al. [69]. These authors have found that anatase is formed only over 400 °C, keeping its amorphousness up to this temperature. Tryba et al. [70] also showed that N-TiO_2_ samples calcinated at 200 °C were amorphous, and those heat-treated at 300–500 °C had only anatase phase. As the annealing temperature increases up to 550 °C, anatase diffraction peaks intensify, confirming the growth of the crystalline (metastable) anatase phase. This means, as expected, that the films crystallinity increases with the heat treatment temperature [70], a fact which can also be correlated with the possibility of N loss with temperature through the formation of nitric oxide (NO). Since NO is a highly volatile gas, its formation and lost will deprive titania-based films from N, reaching a level at which anatase crystallization is no longer inhibited. At this point, N-TiO_2_ films start to behave like a pure TiO_2_ films. In addition, the (TiO_2_)_anatase_ → (TiO_2_)_rutile_ phase transition, often reported in the literature for temperatures above 600 °C in TiO_2−*x*_N*_x_* matrices [69,71], was not observed in our work. Nevertheless, the effect of N in TiO_2_ films crystallization is still controversial. Although, some authors agree that at ca 550 °C, rutile starts to crystallize from anatase [69], others observed the precipitation of anatase phase (in N-TiO_2_ powders) at ca 400 °C, remaining stable up to 700 °C, after which rutile began to appear [72]. Cheng et al. [73] observed a mixed phase composition similar to Degussa P25 (80% anatase and 20% rutile) in N-TiO_2_ films heated treated at 350 °C.

**Figure 8:**
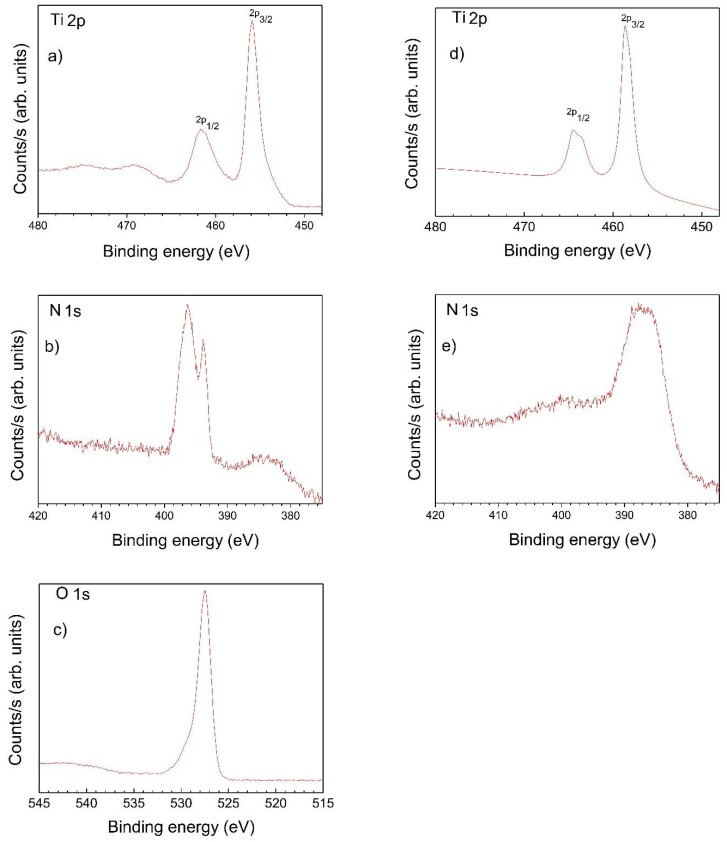
XPS spectra of annealed N-TiO_2_ films at 200 °C: (**a**) Ti 2p, (**b**) N 1s, (**c**) O 1s at 350 °C, (**d**) Ti 2p, and (**e**) N 1s.

In substitutional and/or interstitial solid solution, N may bond to Ti, forming O-Ti-N and/or Ti-O-N bonds [74]. The electron density around Ti atoms increases (through the substitution of O by N), since the N electronegativity (3.04) is lower than that of O (3.44). To confirm the presence of the above bonds, XPS spectra were recorded. Figure 8 shows XPS of N-TiO_2_ films annealed at 200 °C and 350 °C.

In XPS of N-TiO_2_ films, annealed at 200 °C, the binding energies (BE) of Ti 2p_3/2_ and Ti 2p_1/2_ were observed at 461.5 eV 455.9 eV, respectively. A red shifted relative to the pure TiO_2_ values (Ti 2p_3/2_(464.9 eV) and Ti 2p_1/2_(458.8 eV) [75]) is observed, which is attributed to the presence of Ti-O and Ti-N in N-TiO_2_ lattice, confirming two types of bridging anions (Ti-N-Ti and Ti-O-Ti). Details of Ti 2p, O 1s, and N 1s peaks in amorphous N-TiO_2_ XPS spectra at 200 °C are summarized in Table 1. The peaks at 461.5 (Ti 2p_3/2_) and 455.9 (Ti 2p_1/2_) eV in the XPS spectra correspond to Ti^4+^ and Ti^3+^ ions, respectively [75,76,77].

For the same films (N-TiO_2_, annealed at 200 °C) the O 1s core level is centered at 527.9 eV. The main O 1s peak is attributed to oxygen in TiO_2_ matrix (Ti-O-Ti), while the shoulder (at higher binding energy 531.7 eV) is assigned to mixed contributions from surface hydroxides (OH) and carbon oxide compounds, at film surface [78]. N 1s core level for N-TiO_2_ shows a double peak (396.3 and 393.8 eV), indicative of two different N environments. The high binding energy at ca 396.3 eV is assigned to NO or NO_2_ molecules, whereas the low binding energy component at 393.8 eV is attributed to substitutional N in the TiO_2_ lattice, in N-Ti-N bonds [79]. N in Ti-O-N-Ti environment, with binding energy above 400 eV [80], was not observed.

Figure 8 also shows the Ti 2p and N 1s core levels XPS spectra in N-TiO_2_ films, after annealing at 350 °C (in which anatase nanocrystallites precipitated within the a-TiO_2_ matrix). The XPS spectra for the N 1s region exhibit a main peak at around 388.4 eV and a minor one near 400 eV. The appearance of the peak below 395 eV (average of the two values found in the amorphous structure) shows that O-Ti-N bonds have been formed in the TiO_2_ matrix. This indicates that the TiO_2_ structure has been changed by the presence of N atoms around the anatase nanocrystals. The peak at 400 eV could be ascribed to the oxidized nitrogen of Ti-O-N [81]. This is in accordance with the possibility of the formation of nitric oxide (NO) that escapes from the glassy structure as temperature increases. With respect to the Ti 2p binding energy region, compared with those obtained at 200 °C (461.5 eV and 455.9 eV, respectively assigned to Ti 2p_3/2_ and Ti 2p_1/2_), there is a 1.7 eV shift in the electron binding energy toward higher energy. Thus, it is expected that the electron density will increase around the Ti atom, due to N oxidation to NO gas phase, as temperature increases.

The possibility of carbon incorporation (in substitutional solid solution) in the N-TiO_2_ films was discarded by the absence of a low energy peak at 282 eV (attributed to the Ti-C bond, when C substitutes oxygen [82]). Only a low intensity C 1s peak centered at 285 eV was observed in the samples heat-treated at 200 °C. Moreover, this peak disappears with temperature (only traces were observed at N-TiO_2_ films heat-treated at 350 °C), confirming the weakly bonded nature of the C bonding.

**Figure 9:**
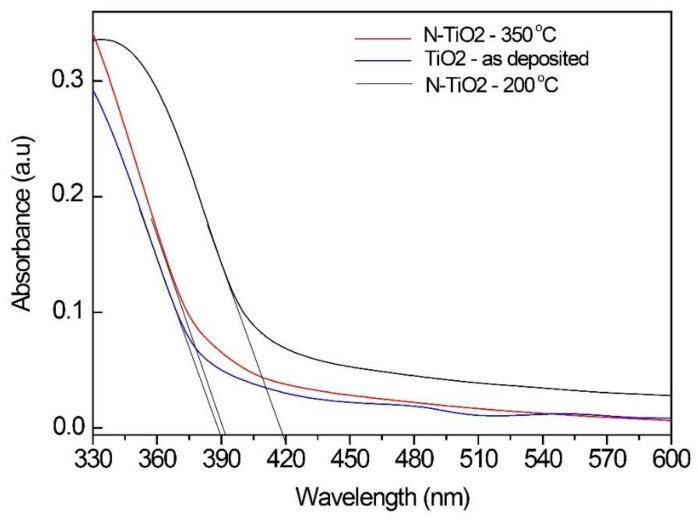
UV-Vis absorption spectra of (blue) a-TiO_2_ film (as-deposited), (black) TiO_0.25_N_0.75_ film heat-treated at 200 °C (amorphous N-TiO_2_), and (red) TiO_0.25_N_0.75_ film heat-treated at 350 °C (anatase nanocrystals precipitated in amorphous matrix).

The UV-vis spectra of a-TiO_2_ film (as deposited and dried) and TiO_0.25_N_0.75_ films heat-treated at 200 °C (amorphous) and 350 °C (with a precipitated phase of anatase nanocrystals) are shown in Figure 9. The straight-line extrapolation method was used to determine the light absorption edge of the samples. The absorption edge of a-TiO_2_ is approximately 390 nm, of anatase is 395 nm, while that of N-doped TiO_2_ is around 415 nm. According to equation

𝐸 = 1240/𝜆 (nm)(5)

the energy of the band gap of a-TiO_2_ (as-deposited) and N-doped TiO_2_ films (heat-treated at 200 °C and 350 °C) are, respectively, 3.18 eV, 2.99 eV, and 3.13 eV. These results show that amorphous pure (as-synthesized) and N-doped TiO_2_ (heat-treated at 200 °C) films exhibit a band gap slightly smaller than anatase (3.2 eV). The presence of anatase nanocrystals within a-TiO_2_ matrix (N-doped TiO_2_ heat-treated at 350 °C) did not largely affected the characteristic optical absorption of a-TiO_2_ films.

The amorphous TiO_2_ film exhibits its characteristic spectrum with the fundamental absorption sharp edge around 390 nm. A red shift is evident in the N-doped TiO_2_ samples, while pure TiO_2_ absorb UV light. There is a minimum band gap value for N-doped TiO_2_ films, heat-treated at 200 °C for 2 h. Higher calcination temperatures, besides delaying titania crystallization, promote a red shift in UV-visible spectra.

### 2.2. Case Study 2. Bactericide Performance of Amorphous TiO_2_-Based NPs

Following industrial demand, bactericide titania NPs were developed to impregnate cotton and polyester textiles for use in hospital environments [83]. Based on our previous work [84], amine was chosen to in situ functionalize titania NPs, due to its exceptional bonding afinity to both cotton and polyester textiles. A novel sol-gel process (room temperature, surfactant-free, and base-catalyzed) was developed for titania NPs production.

**Figure 10:**
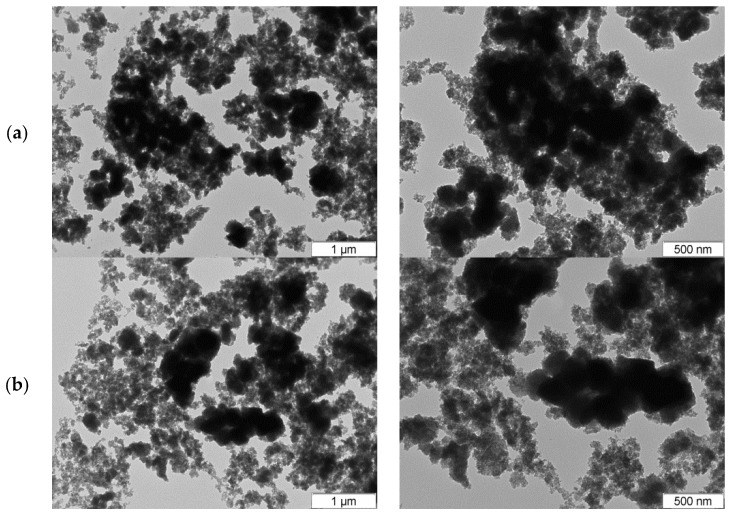
TEM images of a-TiO_2_-based NPs ((**a**) TiO_2_ and (**b**) TiO_2_-NH_2_).

Size and morphology of the synthesized a-TiO_2_-based NPs were analyzed using TEM images. Figure 10a,b shows images of synthesized TiO_2_ and TiO_2_-NH_2_ NPs, respectively. The obtained NPs exhibit a high aggregation tendency and a very small static diameter (<5 nm), which prevent the diameter quantification.

**Figure 11:**
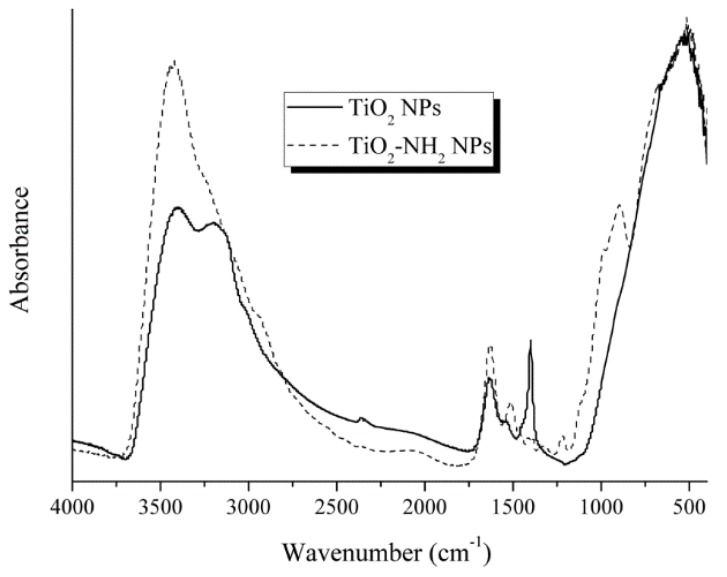
FTIR spectra of a-TiO_2_-based NPs.

In situ amine functionalization of amorphous titania NPs (TiO_2_-NH_2_) was confirmed by FTIR analysis (Figure 11). A shoulder at ~2940 cm^−1^, assigned to C-H stretching, and peaks centered at 1564 cm^−1^, 1482 cm^−1^, 1430 cm^−1^, and 1382 cm^−1^ confirm the presence of ammonium carbamate compounds ([85] and references within), proving the TiO_2_ NPs functionalization with amine group. The main FTIR TiO_2_ peak, centered at 526 cm^−1^, has been assigned to Ti-O bond and is present in all spectra. The TiO_2_-NH_2_ NPs spectrum shows an additional peak centered at 1510 cm^−1^, which is also assigned to Ti-O and Ti-O-Ti framework bonds.

**Figure 12:**
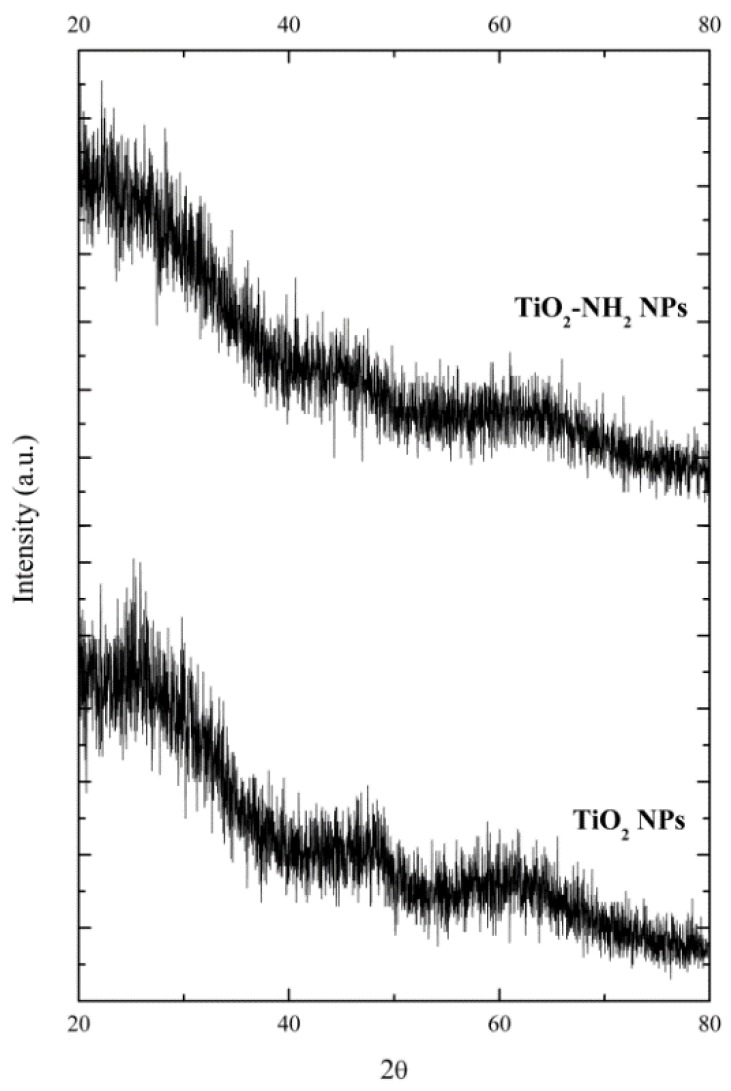
XRD diffractograms of a-TiO_2_-based NPs.

XRD characterization was performed to confirm the amorphous character of the synthesized TiO_2_-based NPs. Diffractograms of TiO_2_-based NPs (TiO_2_ NPs and TiO_2_-NH_2_ NPs) are shown in Figure 12.

The bactericide properties of the a-TiO_2_-based NPs were determined for *Escherichia coli* strain according to ASTM E 2149 (incubation period of 1 h, at room temperature, under daylight). Results are presented in Table 2, and it can be seen that all a-TiO_2_-based NPs exhibit bactericide efficiency above 50%. Statistical analysis using one-way ANOVA, followed by Tukey’s multiple comparison test to determine the significance of the results, showed that the bactericide behavior is not significantly different between different NPs. The bactericide performance could be compared to the crystalline titania NPs behaviour (17 nm diameter and 10, 100, and 500 μg/mL concentration) reported in the literature [85], in which bacterial viability of 100%, 65%, and 20%, respectively, was achieved. It is noteworthy that the less severe experimental conditions were used in the referred work [86]—higher *E. coli* exposition time (24 h instead of 1 h) and UV light (instead of daylight). In our study, bactericide tests were run at daylight (no artificial UV light was used) for much shorter incubation period, proving the bactericide performance of a-TiO_2_ NPs (synthesized at room temperature, without any heat treatment).

## 3. Conclusions

Amorphous solid in which the electronic structure is mainly determined by the short-range neighborhood, in which local order is still predominant, tend to present an electronic band gap, which is slightly smaller than that in corresponding crystalline solids. The existence of this gap is supported by significant and increasing experimental evidence, and several theoretical models have also been developed to explain it. In this work, we observed the same behavior for amorphous titania films. We measured a photonic band gap of 3.18 eV (390 nm) for a-TiO_2_, and 2.99 eV (415 nm) for amorphous N-doped TiO_2_, which was slightly smaller than the value in the literature for crystalline anatase (3.2 eV). This suggests that a-TiO_2_ might be used in the development of optical and electronic devices, replacing more expensive crystalline solids.

Moreover, the relatively low value of this electronic gap allows electrons to jump to the upper band, leaving electronic holes (*h*^+^) in the lower band, which behave as strong oxidants, reacting with oxygen-containing molecules and forming reactive oxidant species (ROS), which in turn are able to disrupt the cellular membrane of simple biological systems, such as bacteria. This was observed in our experimental work, with amorphous sol-gel titania-based NPs acting on *E. coli* bacteria (made according to ASTM E2149), showing that a-TiO_2_ has bactericide potential (even in the absence of daylight and for only 1 h of incubation period), suggesting that it might be used in environmental and medical applications. 

## 4. Materials and Methods

### 4.1. Case Study 1

Titanium IV isopropoxide, Ti(OCH(CH_3_)_2_)_4_ (TiPOT, 97%, Aldrich, Darmstadt, Germany); ethanol (EtOH, 99.9%, Fisher Scientific, Hampton, New Hampshire, EUA); aceylacetone (AcAc, 99%, Fisher Scientific, Hampton, New Hampshire, EUA); and ethylenediamine, C_2_H_4_N_2_H_4_. (EDA, 99%, Fisher Scientific, Hampton, New Hampshire, EUA) were used without any further purification. N-doped TiO_2_ films with a nominal nitrogen doping level of 0.75 (molar ratio of N/TiO_2_) were prepared by sol-gel method as follows: TiPOT (0.02 mol) and ethanol were mixed together and stirred for 30 min and then added to a solution of AcAc. A certain quantity of EDA was then added dropwise under vigorous stirring with the concentration of nitrogen doping of 0.015 mol. The previous solution was then stirred under reflux at 75 °C for approximately 12 h. Subsequently, a few drops of acetic acid (CH_3_CO_2_H) were added for peptidization, and the following hydrolysis process was achieved by adding distilled water drop-wise into the solution under vigorous stirring. Clear and stable yellowish sols were obtained using this process.

In an early stage of the alkoxide reaction with EDA a radical substitution occurs [75], according to the reaction (6):

Ti(OCH(CH_3_)_2_)_4_ + *x* C_2_H_4_N_2_H_4_ → Ti(OCH(CH_3_)_2_)_4−*x*_ (C_2_H_4_N_2_H_3_)*_x_* (6)

Then, the hydrolysis takes place in accordance with the reaction (7):

Ti(OCH(CH_3_)_2_)_4−*x*_ (C_2_H_4_N_2_H_3_)*_x_*+ (4 − *x*) H_2_O → Ti(OH)_4−*x*_(C_2_H_4_N_2_H_3_)*_x_*+ (4 − *x*) (CH_3_)_2_CHO (7)

Finally, a series of condensation reactions occurred to form TiO_2−*x*_N*_x_* and Ti-O(N)-Ti bonds.

The TiO_0.25_N_0.75_ sol-gel films were obtained through deposition of some drops of the sol onto Si (100) wafers by spin coating, at 3000 rpm, for 30 s, using a Laurel Spinner, model WS650, leading to a typical film thickness of approximately 100 nm. The films were dried at 100 °C and then annealed at 200, 350, and 550 °C for 2 h.

The film structure was characterized by Grazing Incidence X-ray Diffraction. For this propose, a Siemens D-5000 diffractometer was used with CuKα radiation. The diffracted intensity was measured at a 2° grazing incidence angle, in the 2θ range between 10° and 40°, with a step size of 0.04°.

The XPS spectra of the films were collected on an ESCALAB 250 spectrometer equipped with dual aluminum-magnesium anodes using a monochromatic Al Kα X-ray radiation (*hν* 1486.6 eV). The XR5 Gun spot used was of 500 μm (15 kV–150 W). The charging effect of these dielectric samples was avoided by flooding the sample with a separate flood gun source of low energy. The high-resolution spectra were carried out after removing the surface contamination by ion sputtering. The sputtering process was performed with a 3 keV Ar^+^ ion beam for 5 min. For all the measurements, pressure in the ultra-high vacuum (UHV) analysis chamber was less than 8 × 10^−9^ mbar, avoiding that the ejected photoelectron interacts with gas molecules. The energy resolution of the survey spectra is 1 eV. The energy resolution of the other spectra is 0.1 eV.

### 4.2. Case Study 2

All chemicals, aqueous sodium silicate solution (SSS) (Na_2_O·SiO_2_, 27% wt % SiO_2_), titanium IV isopropoxide (TiPOT, 97%), *3-aminopropyltriethoxysilane* (APTES, 99%), and tetraethyl orthosilicate (TEOS, ≥99%) were purchased from Sigma-Aldrich (Darmstadt, Germany) and used without further purification. Absolute ethanol (EtOH, 99.5%), from Merck (Darmstadt, Germany), and bi-distilled water (conductivity 0–2 µS/cm^3^, pH 5.8–6.5) were also used. Ammonium hydroxide solution (NH_4_OH, 25% *w*/*w*) was purchased from Scharlau (Barcelona, Spain), and Luria Broth (LB) and Luria Agar (LA) were purchased from VWR.

Amorphous TiO_2_ NPs were synthesized through a sol-gel methodology, at room temperature in less than 1 h and at basic pH. Briefly, a volume of 280 µL of SSS (the nucleating agent) was diluted in 25 mL of absolute ethanol, and the resulting solution was placed under magnetic stirring for 15 min. A mixture of 12.6 mL of ethanol and and 28 mL of ammonium hydroxide was then added to the suspension and stirred for 15 min. After this time, the suspension was placed in the ultrasounds, and 500 µL of titanium isopropoxide (TiPOT) was added, followed by 30 min of sonication.

Amorphous amine-functionalized titania NPs (TiO_2_-NH_2_ NPs) were prepared according to the methodology described for TiO_2_ NPs synthesis (in situ methodology). After the addition of titanium isopropoxide in the ultrasounds, the mixture was sonicated during 30 min. Then, a volume of APTES was added, and the mixture was left in magnetic stirring for 24 h at room temperature. The added volume of TiPOT and APTES was in the molar proportion of TiPOT:APTES—8:2.

Amorphous TiO_2_-based NPs acronyms and compositions are presented in Table 3.

Morphology and size (static diameter) of synthesized TiO_2_ NPs were determined by transmission electron microscopy (TEM). To prepare a TEM sample, a drop of the suspension was placed on a copper grid and dried at room temperature. A Hitachi H-8100 model was used, and the micrographs were obtained using an applied tension of 200 kV. This model is a conventional TEM with a high brightness LaB6 electron source and large specimen-tilt (>30°) capabilities.

The *fingerprints* of the titania-based NPs were performed through Fourier transform infrared spectroscopy (FTIR). FTIR was performed with potassium bromide pellets (KBr, ≥99, FTIR grade, from Sigma-Aldrich). TiO_2_ NPs were finely ground and mixed with potassium bromide, and then pressed into a disc (5 mg TiO_2_-based NPs to 200 mg KBr). KBr pellet was used as background. Nicolet 5700 model was used in transmission mode, through a KBr beamsplitter.

To analyse the crystal structure of synthesized NPs, powder X-ray diffraction (XRD) was used. The diffractograms were obtained with a Malvern PANalytical (Malvern, UK) diffractometer using Cu Kα radiation. Data were collected in steps of 0.02° in the 20–80° range (2θ), with a counting time per step of 4 s.

To determine the bactericide activity, an assay based on the guidelines of the standard test method ASTM E2149 (*Determining the Antimicrobial Activity of Immobilized Antimicrobial Agents Under Dynamic Contact Conditions*) was followed. The amount of TiO_2_-based NPs tested in this assay was 5 mg, which was suspended and incubated in a final volume of 1 mL against the *Escherichia coli* ATCC 2522 (an indicator strain).

Briefly, the assay was performed as follows: the bacterial cells were grown in LB overnight at 37 °C and diluted using a phosphate-buffered saline solution to achieve a concentration of 2.4 × 10^9^ cells mL^−1^. After that, 100 µL of cells was added to the different samples (TiO_2_-based NPs), which were previous suspended in 900 µL of phosphate-buffered saline solution. These suspensions were incubated for 1 h at room temperature and then diluted from 10^0^ to 10^−5^ in NaCl (0.9%). Bacterial reduction was calculated using a standard pour plate technique. Aliquots of each sample were seeded over culture plates with LA medium in triplicates, which were then incubated for 24 h at 37 °C. For the determination of bacterial reduction, the Colony Forming Units (CFU) were counted and the reduction, R (%), was quantified by the expression given in (8). C_0_ represents the CFU of the control sample, and C corresponds to the CFU of the samples with the material intended to analyse.

(8) $$R\;\left(\%\right)\;=\;\frac{\left(C_{0}-C\right)}{C_{0}}\times 100$$

The control assays were performed by incubating bacterial cells in the absence of TiO_2_-based NPs and incubating the NPs without a bacterial performance. No replicates were made.

## Figures and Tables

**Table 1:** XPS details of Ti 2p, O 1s, and N 1s peaks in amorphous N-TiO_2_ films annealed at 200 °C.

	Ti 2p		O 1s		N 1s	
	Ti 2p_3/2_	Ti 2p_1/2_	Shoulder	Main Peak	Peak 1	Peak 2
**BE (eV)**	461.5	455.9	531.7	527.9	396.3	393.8
**Valence states/bond environment**	Ti^3+^ Ti-N	Ti^4+^ Ti-O	O-H	O^2−^	N-O	N-Ti

**Table 2:** Bacterial reduction of *E. coli* achieved by a-TiO_2_-based NPs.

Sample	Bacterial Reduction (%)
TiO_2_ NPs	∼54.07 ± 4.49
TiO_2_-SiO_2_-NH_2_ NPs	∼67.28 ± 9.69

**Table 3:** Amorphous TiO_2_-based NPs: acronyms, compositions, and sol-gel precursor’s volumes.

Acronym	Precursors	V_TiPOT_:V_Prec_ (μL)	Catalyst
TiO_2_ NPs	TiPOT	500	Ammonia solution
TiO_2_-SiO_2_-NH_2_ NPs	TiPOT:APTES 8:2	TiPOT: 417 APTES: 83	Ammonia solution
